# Heterogeneous Streptomycin Resistance Level Among *Mycobacterium tuberculosis* Strains From the Same Transmission Cluster

**DOI:** 10.3389/fmicb.2021.659545

**Published:** 2021-06-11

**Authors:** Deisy M. G. C. Rocha, Carlos Magalhães, Baltazar Cá, Angelica Ramos, Teresa Carvalho, Iñaki Comas, João Tiago Guimarães, Helder Novais Bastos, Margarida Saraiva, Nuno S. Osório

**Affiliations:** ^1^Life and Health Sciences Research Institute (ICVS), School of Medicine, University of Minho, Braga, Portugal; ^2^ICVS/3B’s – PT Government Associate Laboratory, Braga, Portugal; ^3^i3S – Instituto de Investigacão e Inovação em Saúde, University of Porto, Porto, Portugal; ^4^Instituto de Biologia Molecular e Celular (IBMC), University of Porto, Porto, Portugal; ^5^Department of Clinical Pathology, Centro Hospitalar São João, Porto, Portugal; ^6^Biomedicine Institute of Valencia IBV-CSIC, Valencia, Spain; ^7^CIBER in Epidemiology and Public Health, Valencia, Spain; ^8^Institute of Public Health, University of Porto, Porto, Portugal; ^9^Department of Biochemistry, Faculty of Medicine, University of Porto, Porto, Portugal; ^10^Serviço de Pneumologia, Centro Hospitalar Universitário de São João EPE, Porto, Portugal

**Keywords:** tuberculosis, streptomycin, resistance, transmission, evolution

## Abstract

Widespread and frequent resistance to the second-line tuberculosis (TB) medicine streptomycin, suggests ongoing transmission of low fitness cost streptomycin resistance mutations. To investigate this hypothesis, we studied a cohort of 681 individuals from a TB epidemic in Portugal. Whole-genome sequencing (WGS) analyses were combined with phenotypic growth studies in culture media and in mouse bone marrow derived macrophages. Streptomycin resistance was the most frequent resistance in the cohort accounting for 82.7% (*n* = 67) of the resistant *Mycobacterium tuberculosis* isolates. WGS of 149 clinical isolates identified 13 transmission clusters, including three clusters containing only streptomycin resistant isolates. The biggest cluster was formed by eight streptomycin resistant isolates with a maximum of five pairwise single nucleotide polymorphisms of difference. Interestingly, despite their genetic similarity, these isolates displayed different resistance levels to streptomycin, as measured both in culture media and in infected mouse bone marrow derived macrophages. The genetic bases underlying this phenotype are a combination of mutations in *gid* and other genes. This study suggests that specific streptomycin resistance mutations were transmitted in the cohort, with the resistant isolates evolving at the cluster level to allow low-to-high streptomycin resistance levels without a significative fitness cost. This is relevant not only to better understand transmission of streptomycin resistance in a clinical setting dominated by Lineage 4 *M. tuberculosis* infections, but mainly because it opens new prospects for the investigation of selection and spread of drug resistance in general.

## Introduction

Emergence of drug-resistant *Mycobacterium tuberculosis* strains, classically attributed to lack of patient adherence to the prolonged therapeutic regimens, hinders tuberculosis (TB) control (Murray et al., 2015; WHO, 2020). Resistant *M. tuberculosis* strains can transmit in the population, therefore amplifying the drug resistance (DR) problem (Becerra et al., 2019). Thus, understanding the mechanisms and molecular bases underlying DR acquisition and transmission is key to overcome a most challenging issue in TB treatment.

Resistance to streptomycin (STR), an antibiotic used in the past as a TB monotherapy, was the first *M. tuberculosis* DR described (Pyle, 1947; Crofton and Mitchison, 1948). Although STR is now a second-line TB medicine, STR resistant *M. tuberculosis* isolates remain frequent, with a special high incidence in Western-Europe (Manson et al., 2017). In Germany, monoresistance to STR was both the most prevalent form of DR and the most frequent DR among multidrug-resistant (MDR) stains (Glasauer et al., 2019). In Portugal, mutations in STR target genes were identified in isolates from the Lisbon family genetic cluster Q1, a cluster associated with MDR in the Lisbon area (Perdigão et al., 2013, 2014). Furthermore, a whole genome-based study suggested isoniazid and STR resistance mutations as precursors of MDR *M. tuberculosis* strains (Manson et al., 2017). The sustained transmission of STR resistant strains, as well as its possible implications for MDR acquisition, should not be neglected, calling for more studies in this area.

STR resistance is primarily associated with mutations in *rpsL*, *rrs*, and *gid*, genes, which, respectively, encode for the components of the 30S ribosome subunits S12, 16S rRNA and for a 7-methylguanosine methyltransferase specific for the 16S rRNA (Okamoto et al., 2007; Jagielski et al., 2014). The mutated gene might modulate the level of STR resistance. Polymorphisms in the *rpsL* gene were associated to high STR resistance levels, while those in the *rrs* locus or in the *gid* gene were linked to intermediate to high and low STR resistance levels, respectively (Wong et al., 2011; Smittipat et al., 2016; Cohen et al., 2020). The type of STR resistant mutation seems to vary according to *M. tuberculosis* phylogeography. For example, specific mutations in *rpsL* or *rrs* (*rpsL*:Lys43Arg, Lys88Arg; *rrs*: a514c or c517t) are very common among the STR-resistant isolates from South-East Asian countries with high incidence of lineage 2 (Nhu et al., 2012; Smittipat et al., 2016; Hlaing et al., 2017). In contrast, polymorphisms in *gid* were more associated with STR resistance in isolates from Euro-American lineage 4 (L4) (Hlaing et al., 2017; Oppong et al., 2019). The high frequency of STR resistance in Western-Europe may therefore result from the high prevalence of STR resistant mutations in *gid* and their efficient transmission in the absence of antibiotic pressure. Here, we retrospectively studied the STR resistance prevalence in a previously reported cohort of 681 individuals (Bastos et al., 2016; Sousa et al., 2020), from a TB epidemic in Portugal, and used genomics and culture models to compare STR resistant and susceptible clinical isolates in terms of inferred transmissibility, genetic diversity, and growth.

## Materials and Methods

### Bacterial Culture and Drug Susceptibility Tests

A cohort of 681 culture-confirmed TB cases diagnosed at Centro Hospitalar São João (CHUSJ), Porto-Portugal between 2007 and 2013 was used (Bastos et al., 2016; Sousa et al., 2020). Isolates were obtained from the sputum of the patients and then cultured in a biosafety level 3 facility on solid 7H11 agar supplemented with Oleic Albumin Dextrose Catalase Growth Supplement (OADC) and plymyxin B, amphotericin B, nalidixic acid, trimethoprim and azlocillin (PANTA antibiotic mixture, BD). The plates were incubated for 3 weeks at 37°C. After this period, colonies were suspended, optical density was adjusted to 0.01–0.03 (OD620nm) and BACTEC^TM^ MGIT^TM^ 960 tubes (Mycobacteria Growth Indicator Tubes, BD Biosciences) supplemented with OADC were inoculated and grown until mid-log phase. These bacterial cultures were subcultured for drug susceptibility testing (DST) to isoniazid (INH), rifampicin (RIF), STR, ethambutol (ETH), and pyrazinamide (PZA) using a protocol based on the proportion method (MOP) (Woods et al., 2011). Briefly, a 7.0 mL inoculum was prepared in a BD MGIT tube and used after positivity to inoculate a new BD MGIT tube with an antibiotic. In each tube, 800 μL of BD BACTEC MGIT SIRE Supplement (Middlebrook OADC) was aseptically added. In the drug-containing tube it was added 100 μL of antibiotic and then 500 μL of positive inoculum. In the growth control tube 100 μL of *M. tuberculosis* suspension was diluted in 10 mL of saline solution, then 500 mL of the diluted suspension was transferred into a BD MGIT suspension. The concentration used was the recommended critical concentration for MOP, 1.0 mg/L STR; 0.1 mg/L INH; 1.0 mg/L RIF; 5.0 mg/L ETH. For STR and INH a higher concentration was used to profile the degree of resistance, 4.0 and 0.4 mg/L, respectively. The isolates were considered resistant if 1% or more of the test population grew in the presence of the critical concentration. Monoresistance was defined as resistance exclusively to one of the four first-line anti-tuberculosis drugs tested. Polyresistance was defined as resistance to two or more anti-tuberculosis drugs, but not both isoniazid and rifampicin. MDR was resistance to at least isoniazid and rifampicin simultaneously.

### Streptomycin Resistance Level

Mid-log phase cultures were used to test the phenotypic susceptibility to streptomycin. 500 μL of the bacterial cultures were inoculated in BACTEC^TM^ MGIT^TM^ 960 tubes (BD Biosciences) supplemented with 800 μL of OADC and 100 μL of STR. The cultures were incubated at 37 °C until the stationary phase of growth or a maximum of 42 days. The concentrations used to determine the resistance level were 1.0 and 10.0 mg/L of STR. For each *M. tuberculosis* clinical isolate, a drug-free control (*M. tuberculosis* only) was also included. Bacterial cultures were stopped at the planktonic growth phase or after 42 days of incubation. The BACTEC growth measurements were reported automatically. Resistance level was defined as high if the growth was observed at both STR concentrations with the same time to positivity, intermediate if the bacteria grew at both concentrations with high time to positivity at the highest STR concentration, and as low if the bacteria grew only at the lowest STR concentration.

### BMDM Generation and Infection

Macrophages were derived *in vitro* from mouse bone marrow cell suspensions as previously described (Moreira-Teixeira et al., 2016; Bhatt et al., 2018). On day 7, cells were harvested, counted, plated in 96 well plates and infected with a MOI of 2. Four hours post-infection the cells were washed and incubated without STR or with 10.0 and 100.0 mg/L of STR. Six days post-infection macrophages were lysed with saponin and the number of viable bacteria determined by CFU enumeration.

### Whole-Genome Sequencing and Analysis

DNA was extracted using the phenol-chloroform/bead beater method as described previously (Sousa et al., 2020). Paired-end sequencing of the DNA extracted from each isolate was performed in Illumina MiSeq, HiSeq 2000/2500 and NextSeq 500 instruments. Raw reads were subjected to quality trimming with Trimmomatic v.0.38 (Bolger et al., 2014) (minimum read length 20 and average base quality 20 in 4-base sliding windows) and quality control with FastQC v.0.11.7 (Andrews, 2010) and MultiQC v1.0 (Ewels et al., 2016). Filtered reads were mapped to the inferred MTBC ancestral strain (Comas et al., 2010) with bwa-mem (Li, 2013) and converted to BAM files with samtools v.1.3.1 (Li et al., 2009). Duplicated reads were removed with Picard MarkDuplicates v.2.18.14 (Broad Institute, 2018). Genomes with more than 5% of missing sites were excluded. High confidence single nucleotide polymorphisms and insertions/deletions were called with Pilon (Walker et al., 2014) v.1.22 (minimum depth of five valid read pairs, minimum mapping quality 20 and minimum base quality 20) and filtered with bcftools v.1.3.1 (Li, 2011) (keeping only biallelic sites with variant frequency ≥ 75% and with a minimum distance from indels of four nucleotides). Genomic variants were annotated with SnpEff (Cingolani et al., 2012) *M. tuberculosis* H37Rv reference genome annotation (NC_000962.3; GCF_000195955.2). To minimize false positives, previously identified repetitive genomic regions, mobile elements and genes containing ≥ 50 bp nucleotide chunks identical to other parts of the genome (Coscolla et al., 2015; Koch et al., 2017) were not considered to further analysis. Raw reads were also analyzed using TBProfiler v.2.8.12 with the database from 2nd of April of 2020.

### Phylogenetic Analysis

The high-confidence polymorphisms identified for each isolate were inputted into sequences from the inferred MTBC ancestral using bcftools v.1.3.1 (Li, 2011) consensus. The resulting whole-genome sequences were aligned using MAFFT v.7 (Katoh and Standley, 2013). Phylogenetic analysis was performed from the obtained multiple sequence alignment using IQ-TREE v.1.5.5 (Nguyen et al., 2015). The model TVM + R2 was used since it was the best-fitting model according to AIC, as determined by ModelFinder (Kalyaanamoorthy et al., 2017). Ancestral state reconstruction was done on the obtained rooted tree with log likelihood restricted maximum *a posteriori* (MAP) using the binary classification of each sequence as STR resistant or susceptible following the results from the performed phenotypic tests. The visual representations of the trees were done with FigTree v.1.4.3 (Rambaut, 2009) or PastML v.1.9.24 (Ishikawa et al., 2019). The transmission clusters were defined using a threshold of 12 or less pairwise SNPs between sequences as previously defined (Walker et al., 2013). The minimum spanning tree was performed with Phyloviz (Nascimento et al., 2017).

### Statistical Analysis

Data from culture and drug susceptibility assays were analyzed using GraphPad Prism software, version 8.1.0 and checking for normality and log normality. Student’s *t*-test was used to determine differences between two different groups and One-way ANOVA for more than two groups. Tests were applied as referred in figure legends. To compare the proportions of genomic variants in STR resistant isolates and STR susceptible isolates, Fisher exact test was performed with the fisher.test command R 3.6.3 (R Core Team, 2014), followed by Bonferroni correction. Differences were considered significant for *p* ≤ 0.05 and represented as follows: ^∗^*p* < 0.05; ^∗∗^*p* < 0.01; ^∗∗∗^*p* < 0.001; and ^****^p < 0.0001.

## Results

### Characterization of the STR Resistance Prevalence in the Study Cohort

**FIGURE 1:**
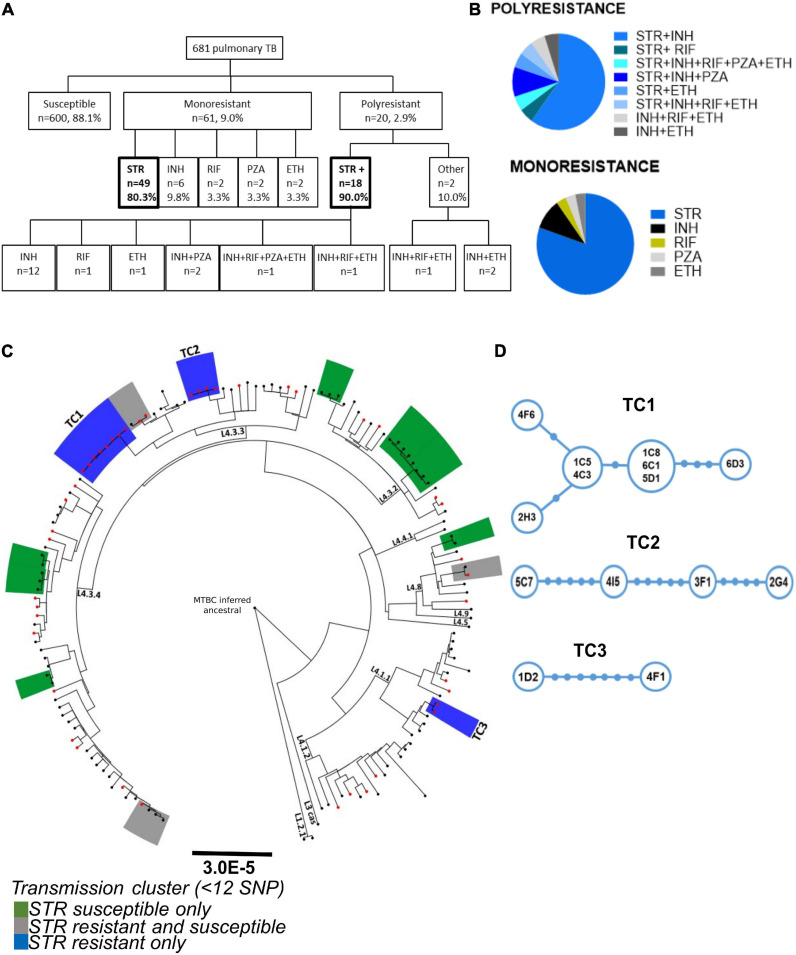
Characterization of the prevalence and transmission of STR resistance in the study cohort. **(A)** Data from the drug susceptibility test of 681 pulmonary TB patients from the São João Hospital cohort (CHSJ). Susceptible refers to strains which displayed no growth in the drug-susceptibility test; monoresistance was defined as resistance exclusively to one of the anti-tuberculosis drugs tested; polyresistance corresponds to resistance to two or more anti-tuberculosis drugs, but not both isoniazid and rifampicin simultaneously; multidrug-resistance (MDR), resistance to isoniazid and rifampicin simultaneously, and/or other anti-TB drugs tested. Boxes in bold highlight the number/percentage of the monoresistant STR resistant isolates and of the polyresistance including STR resistance. STR+, resistance to streptomycin and other(s) anti-TB drug; Other, polyresistance which does not include the STR resistance. STR, streptomycin; INH, isoniazid; RIF, rifampicin; PZA, pyrazinamide; ETH, ethambutol. **(B)** Pie charts with the percentage of monoresistance and polyresistance of the cohort. **(C)** Phylogeny and transmission clusters of *M. tuberculosis* isolates from the CHSJ. Boxes highlighted in blue, green, and gray represent the transmission clusters. Blue, STR resistant isolates only (TC1, TC2, and TC3); green: represent clusters with STR susceptible isolates only and gray, with STR resistant and susceptible isolates. The black dots and red dots indicate the STR susceptible isolates and the STR resistant isolates, respectively. **(D)** Minimum Spanning Tree representation of transmission clusters, TC1, TC2, and TC3. Blue circles represent a node of *M. tuberculosis* isolates. The size of the circle is proportional to the number of strains represented. Filled blue circles in the connections indicate the number of SNPs between the nodes.

A phenotypic drug susceptibility test for INH, RIF, STR, ETH, and PZA was performed in the cohort as part of the routine diagnostic procedure. The analysis shows that 88.1% (*n* = 600) of the *M. tuberculosis* isolates in the cohort were susceptible to all tested antibiotics, while 9.0% (*n* = 61) of the isolates were resistant to only one antibiotic and 2.9% (*n* = 20) displayed resistance to more than one drug (Figure 1A). STR resistance accounted for 82.7% (*n* = 67) of the DR *M. tuberculosis* isolates, being present in 80.3% (*n* = 49) of the monoresistant isolates (Figures 1A,B). This was followed by monoresistance to INH, with 9.8% (*n* = 6) and by RIF, PZA, or ETH with 3.3% (*n* = 2) (Figures 1A,B). Furthermore, 90% (*n* = 18) of the polyresistant isolates also harbored STR resistance (Figure 1A). Finally, 15% (*n* = 3) of the polyresistant isolates were MDR strains, two of which harbored STR resistance (Figure 1B). These results are in line with a previous report in Western-European TB cohorts showing STR resistance as the dominant resistance with approximately 45.8% (*n* = 33/72), followed by INH resistance with 25% (*n* = 18/72) (Manson et al., 2017).

### Evidence for Ongoing Transmission of STR Resistant *Mycobacterium tuberculosis*

The high number of STR resistant *M. tuberculosis* isolates found in this and other cohorts, in which STR is not used presently as first line TB treatment, argues in favor of transmission of these isolates in the population. To investigate this, we inspected the transmission profile of the STR resistant *M. tuberculosis* isolates on the cohort. The whole genomes of 149 *M. tuberculosis* clinical isolates (45 STR resistant strains and a set of 104 randomly selected STR susceptible isolates) were sequenced and a phylogenetic analysis was performed. By defining transmission clusters using a cutoff of 12 or fewer single nucleotide polymorphisms (SNPs) of distance (Walker et al., 2013), we identified a total of 13 transmission clusters (Figure 1C). Most of the transmission clusters (10 of 13) were composed of strains from L4 sublineage LAM (4.3), the most prevalent in this cohort in agreement with our previous study (Sousa et al., 2020). Three transmission clusters were entirely composed of STR resistant isolates, three included both STR resistant and susceptible isolates and seven contained susceptible isolates only (Figure 1C). The isolates in the STR resistant transmission clusters (TC1-3) showed pairwise distances varying from one to seven SNPs (Figure 1D). TC1 is the cluster with the highest number of strains (*n* = 8) and the one harboring the lowest maximum pairwise genetic distance (five SNPs; Figure 1D). These data strongly support that at least certain STR resistant *M. tuberculosis* strains are being transmitted in the studied cohort, thus contributing to the maintenance of this DR phenotype in the population.

### Heterogeneous Growth Rates in the Presence of STR Among *Mycobacterium tuberculosis* Isolates From the Same Transmission Cluster

**FIGURE 2:**
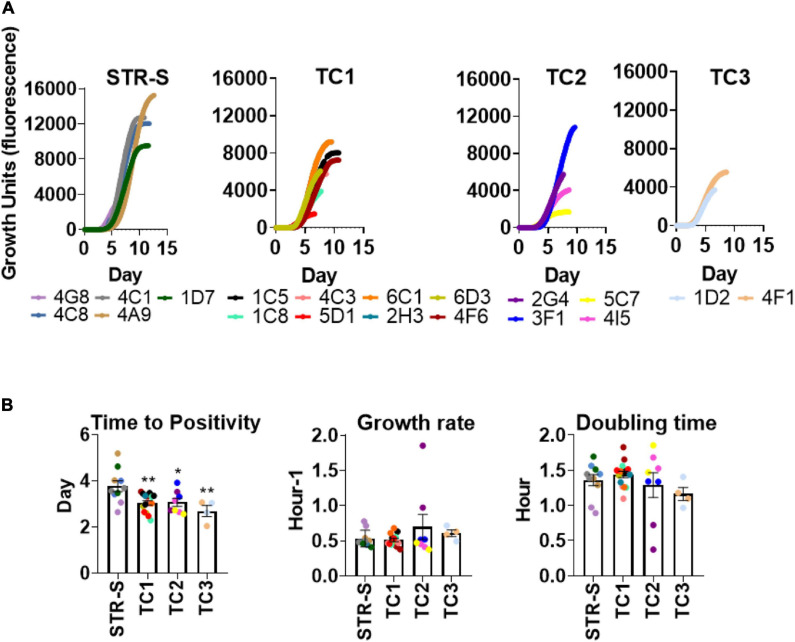
*In vitro* growth comparison of STR resistant and susceptible *Mycobacterium tuberculosis* clinical isolates. **(A)** Growth of *M. tuberculosis* isolates in MGIT^TM^ 960 tubes. Each curve illustrates the average growth of the isolate inoculated in the MGIT culture media, two replicates were included. STR-S, streptomycin susceptible isolates; TC1: T. cluster 1; TC2: T. cluster 2 and TC3: T. cluster 3. **(B)** Comparison of time to positivity, growth rate, and doubling time of different clinical isolates. Data represent the mean and SEM values (*n* = 2 for each strain). Statistical analysis was performed using one-way ANOVA (*p < 0.05 and**p < 0.01).

**FIGURE 3:**
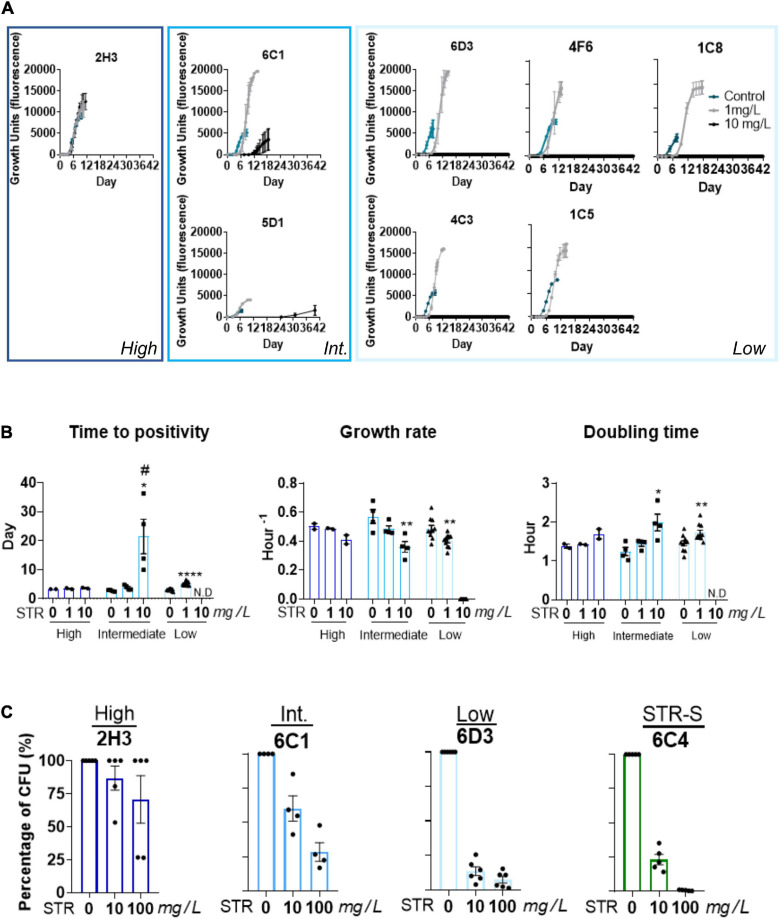
Heterogeneous growth rates in the presence of STR among strains from the same transmission cluster. **(A)** Comparison of the growth of *M. tuberculosis* isolates within TC1 (2H3, 6C1, 5D1, 6D3, 4F6, ICS, 4C3, and 1C5), in absence or presence of STR. The concentrations used were 1.0 mg/L. (gray curve) and 10.0 mg/L (black curve) of STR. The condition without STR was used as the control, represented with a blue curve. **(B)** Comparison of the growth parameters, time to positivity; growth rate, and doubling time of the strains with different STR resistance levels. Low STR resistance level (light blue) intermediate STR resistance level (blue), and high STR resistance level (dark blue). Time to positivity: the time needed to achieve the threshold of 75 GU in the BACTEC MGIT 960 system, growth rate of each isolate, doubling time: the time required for the bacteria density duplicate. The growth rates and doubling times were calculated in the exponential phase of growth. N.D means not detected. The error bar represents SEM values (*n* = 2 for each isolate). Statistical analysis was performed using Student’s *t*-test or one-way ANOVA. Significant statistical differences comparing 0 mg/L of STR to the other conditions are represented by *, and to 1 mg/L of STR by #(# or, *p < 0.05; **p < 0.01; ***p < 0.001; and ****p < 0.0001). **(C)** Percentage of survival of the intracellular bacteria after incubation with 10.0 or 100.0 mg/L of STR. The intracellular bacteria were quantified by CFU enumeration six days post-infection. Representation of the TC1. STR resistant strains with high STR resistance level (dark-blue), STR resistant isolates with intermediate resistance level (blue), STR resistant isolate with low resistance level (light-blue). STR-S refers to STR susceptible isolate (green).

Given that the STR resistant isolates from TC1-3 were likely being transmitted in the population, we hypothesized that these isolates had unimpaired fitness. Since *in vitro* growth capacity is a commonly used surrogate for bacterial fitness (Castro et al., 2020; Degiacomi et al., 2020), we compared the *in vitro* growth profiles of the *M. tuberculosis* isolates in TC1-3 to those of a group of susceptible isolates from the same TB cohort (Figure 2A). *M. tuberculosis* isolates in TC1-3 presented lower times to positivity than the STR susceptible isolates, but similar growth rates and doubling times (Figure 2B). We next evaluated the *in vitro* growth profile of *M. tuberculosis* isolates in TC1, as this cluster included a higher number of STR resistant isolates, in the presence of two doses of STR: 1.0 or 10.0 mg/L. Despite the high genetic relatedness between the isolates in TC1, the addition of STR to the culture medium resulted in distinct growth profiles across the tested isolates. Isolate 2H3 showed the highest STR resistance level, displaying a growth profile that was independent of the dose of STR used (Figure 3A). Isolates 6C1 and 5D1 grew at both concentrations of STR but displayed a delayed growth with significantly higher times to positivity, reduced growth rates and increased doubling times in the presence of 10.0 mg/L of STR (Figures 3A,B). Finally, isolates 6D3, 4F6, 1C5, 4C3, and 1C8 did not grow at the highest STR dose, showing altered growth rates and doubling times in the presence of 1.0 mg/L of STR (Figures 3A,B).

We next questioned whether these differences in STR resistance were only seen in *in vitro* axenic medium, or if they prevailed during macrophage infection. For that, mouse BMDM were infected with isolates 2H3, 6C1, or 6D3 showing, respectively, high, intermediate or low STR resistance levels, and the intracellular bacterial growth quantified by CFU enumeration 6 days post-infection. A STR susceptible isolate (6C4) was included as a control. Similar findings were observed upon infection of BMDM and MGIT *in vitro* growth. Whereas the isolate 2H3 grew intracellularly at all tested concentrations (Figure 3C), isolate 6C1 grew at both concentrations of STR, but showed growth reduction as compared with the condition without STR (Figure 3C). This reduction was more pronounced at 100.0 mg/L of antibiotic. In turn, isolate 6D3 showed significant growth impairment in the presence of both concentrations of the antibiotic (Figure 3C). As expected, the STR susceptible isolate grew normally only in the absence of antibiotic and presented approximately 65–85% growth reduction on bacterial load in the presence of 10.0 mg/L of STR and 99% growth reduction at 100.0 mg/L of STR (Figure 3C). The internal bacterial load of each isolate without STR is represented in the Supplementary Figure 1.

Overall, these findings suggest that the *M. tuberculosis* isolates within TC1 have similar *in vitro* growth capacity, but different STR susceptibility levels which are observed both in axenic medium and during intracellular macrophage infection.

### *Mycobacterium tuberculosis* Isolates Found in Transmission Clusters Harbor Novel STR Resistance Mutations

**FIGURE 4:**
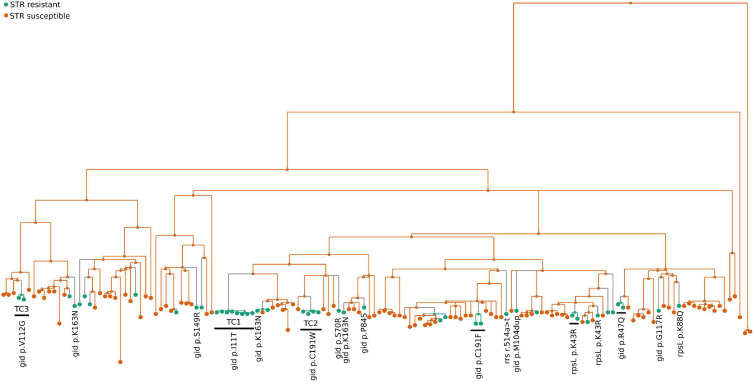
Ancestral state reconstruction (ASR) of STR resistant *M. tuberculosis* isolates. ASR was done using log likelihood restricted maximum *a posteriori* (MAP) analysis of the phylogenetic tree with the binary classification of each sequence as STR resistant (green circles) or susceptible (orange circles) following the results from the phenotypic tests. Previously known *rrs* and *rpsL* STR resistance mutations (*rrs* r.514a>t, *rpsL* p.Lys43Arg and p.Lys88Gln) and *gid* mutations (*gid* p.Arg47Gln, p.Ser70Arg, p.Pro84Ser, p.Met104dup, p.Va1112Gly, p.Ser149Arg, p.Gly117Arg and p.Lys163Asn) that exclusively present in STR resistant isolates are indicated.

**FIGURE 5:**
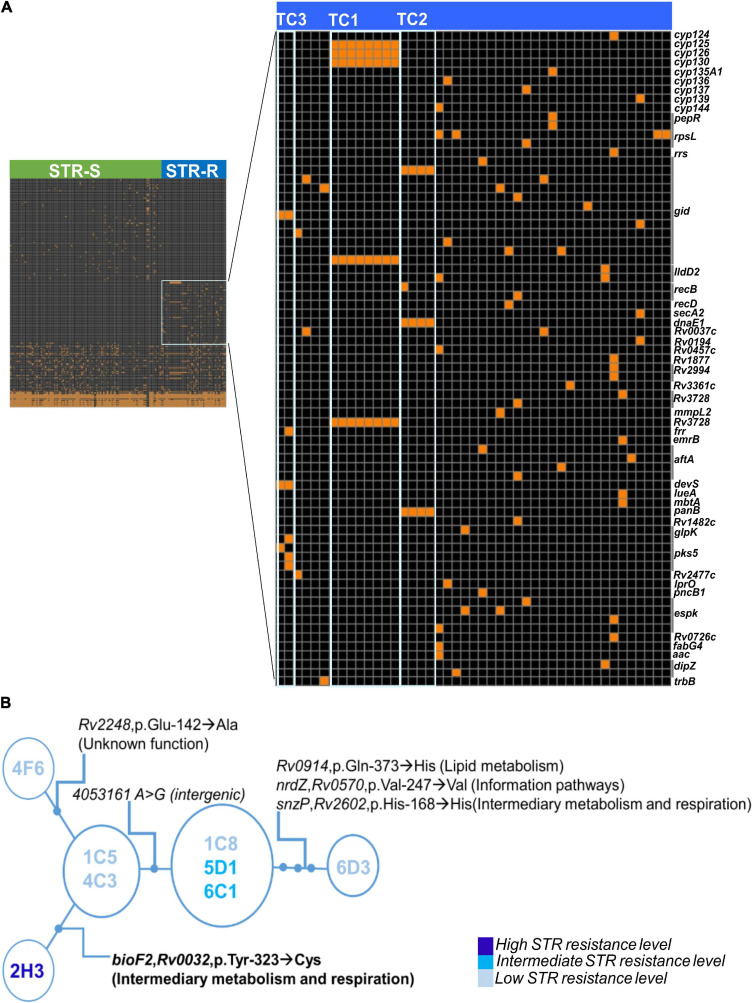
Whole-genome sequencing analysis unveils novel candidate STR resistance mutations. **(A)** Genome-wide analysis of 149 *M. tuberculosis* clinical isolates, 104 STR susceptible and 45 STR resistant strains. The header symbolizes the *M. tuberculosis* isolates, green represent the STR susceptible isolates (STR-S) and blue STR resistant isolates (STR-R). The right axis includes 150 polymorphisms. The black area of the heat map corresponds to the absence of polymorphisms and the orange squares are shown when the polymorphism (SNP or Indel) is found in the different isolates. The area highlighted with a square in light-blue shows the polymorphisms identified exclusively in STR resistant isolates. In the heat map with the STR resistant exclusive polymorphisms, gray line group the polymorphisms presented in the same gene, size of the line depends on the number of SNPs or Indels. Polymorphisms of TC1, TC2, and TC3 are highlighted in the heat map with the light blue boxes. **(B)** Minimum Spanning Tree Representation of cluster TC1 clinical isolates indicating the polymorphisms separating the nodes and the level of STR resistance of each isolate. SNP = single nucleotide polymorphism and Indels = insertions or deletions.

To investigate the genetic bases of STR resistance we started by analyzing the whole genomes of the 149 *M. tuberculosis* clinical isolates using TBProfiler (Phelan et al., 2019). The results corroborated only part of the results of the phenotypic drug susceptibility tests (Supplementary Table 1). Six STR resistant *M. tuberculosis* clinical isolates, not found in transmission clusters, harbored described STR resistance mutations (*rpsL* p.Lys-88→Gln, *rpsL* p.Lys-43→Arg, or *rrs* r.514 a→t) and were detected by both methods. However, the clinical isolates belonging to TC1-3 were incorrectly classified by TBProfiler as susceptible (Supplementary Table 1), suggesting unknown genetic bases for STR resistance. Thus, we performed whole-genome analysis to compare all SNPs and indels between the 45 STR resistant and the 104 STR susceptible isolates. We found a total of 11,516 high confidence polymorphisms (SNPs or indels, Supplementary Figure 2). These included 1,693 polymorphisms that were exclusively found in STR resistant isolates but a statistical association to STR resistance was not found (Supplementary Table 2). This was likely explained by the dispersion in different underlying genetic basis for the multiple introductions of STR resistance in the study cohort. Indeed, the ancestral state reconstruction of STR resistance in the study cohort suggests 30 independent events of STR resistance acquisition that were likely associated with different mutations including several novel *gid* mutations found only on STR resistant isolates (Figure 4). Also, some genetic variants previously associated with low level STR resistance, including *gid* (p.Val-124→Gly) (Bouziane et al., 2019), were in fact found in our cohort in both STR susceptible and resistant *M. tuberculosis* isolates and are unlikely to account to STR resistance. To further characterize possible candidate polymorphisms of drug resistance we selected all polymorphisms that were in previously described *M. tuberculosis* DR genes (Supplementary Figure 2). This allowed narrowing to 75 polymorphisms that were exclusively present in resistant isolates (Supplementary Table 3). Among these SNPs and indels, the ones found in inferred transmission clusters entirely composed of STR resistant isolates (TC1-3) were heterogeneous (Figure 5A). Notably, TC1 shared three non-synonymous SNPs (nsSNP) on Cytochrome p450 (p.Ile-359→Thr, p.His-353→Arg, and p.Ile-344→Val in *cyp125*, *cyp126*, and *cyp130*, respectively), one nsSNP in *gid* (p.Ile-11→Thr) and one nsSNP on a probable conserved transporter from the Major Facilitator Superfamily (p.Arg-798→Cys, *Rv3728*). The isolates from TC2 shared three nsSNPs (*gid* p.Cys-191→Trp, *dnaE1* p.Ser-4Pro, and *panB* p.Gly-211→Ser). Finally, in TC3 all isolates shared two nsSNPs (*gid* p.Val-112Gly and *devS* p.Leu-381Phe). The mutations found in *gid* (p.Ile-11→Thr, p.Cys-191→Trp and p.Val-112→Gly) were most likely underlying STR resistance in TC1-3 although a contribution of other mutations shared in the cluster and not present in susceptible clinical isolates could not be excluded.

We next investigated genetic variants that might impact on the different STR resistance levels within TC1 (Figure 5B). The TC1 isolate with the highest level of STR resistance (2H3) had one nsSNP (p.Tyr-323→Cys) in the gene *bioF2* that was not present in the other TC1 isolates. The gene *bioF2* (*Rv0032*) is likely involved in biotin biosynthesis. *bioF2* p.Tyr-323→Cys was not found in any other isolates of the cohort, but a nsSNP in an adjacent position of *bioF2* (p.Ile-322→Val) was present in another STR resistant isolate of the cohort (4E9). These substitutions were not included in known *bioF2* functional sites. We then investigated the presence of these *bioF2* SNPs in an unrelated database of genomes from 1,450 laboratory confirmed STR resistant clinical isolates and 1,450 susceptible strains from PATRIC (Wattam et al., 2014). The SNPs p.Tyr-323→Cys and p.Ile-322→Val in *bioF2* were not found in any of the queried sequences. The remaining polymorphisms defining the isolates of TC1 were identified in genes with different categorical functions, such as lipid metabolism, cell wall and cell process, or unknown hypothetical proteins (Figure 5B). Except for the *bioF2* nsSNP none of the other SNPs matched the phenotypic differences in STR resistance found among the TC1 strains (Figure 5B). Indeed, isolates 1C8, 5D1 and 6C1 showed mild differences in growth in the presence of STR and did not harbor any high confidence polymorphisms of difference [minimum depth ≥ 5, minimum quality 20, variant frequency ≥ 75% and not located on repetitive genomic regions or mobile elements (Coscolla et al., 2015; Koch et al., 2017)]. Thus, we next analyzed polymorphisms with lower variant frequency (<75% of the reads) to investigate less stable mutations. The analysis highlighted a possible mutation in 1C8 in the homopolymeric tract of seven cytosines of the *glpK* gene, a result that was supported by PCR amplification and resequencing of the samples by the Sanger method (Supplementary Figure 3). In all the studied isolates this mutation was found in two isolates with low STR resistance from TC1 (1C8, 6D3) and two STR susceptible genome sequences (4H7, 5D4). Further studies are needed to investigate if mutations in *glpK* could be related to the observed phenotypes. Collectively, our results highlight novel candidate genetic variants for STR resistance.

## Discussion

Recent data reinforces that transmission of DR strains evolving from mono- to multi-antimicrobial resistance phenotypes fuels the threat of antimicrobial resistance (Al-Mutairi et al., 2019; Becerra et al., 2019; Khan et al., 2019; Knight et al., 2019). Elusively, STR resistance remains the most common type of resistance found in *M. tuberculosis* clinical isolates collected from several parts of the world (Manson et al., 2017), even though the use of STR in TB treatment was demoted.

Development of STR resistance in *M. tuberculosis* is often associated with mutations in *rpsL*, *rrs*, and *gid* genes (Okamoto et al., 2007; Jagielski et al., 2014; Khosravi et al., 2017; Islam et al., 2020; Shrestha et al., 2020). The type of DR mutation might associate with the *M. tuberculosis* lineage and influence the resistance level and fitness (Spies et al., 2011; Ballif et al., 2012; Miotto et al., 2017; Macedo et al., 2018; Sun et al., 2018). The relative fitness of drug resistant vs. susceptible strains has been highlighted as a key factor driving the transmission of DR *M. tuberculosis* (Ballif et al., 2012; Morcillo et al., 2014; Knight et al., 2015). In agreement, we found no growth defect in the STR resistant clinical isolates belonging to the inferred transmission clusters suggesting a normal fitness and likelihood of transmission. Interestingly, the clinical isolates transmitted in this cohort harbored several novel candidate STR resistance mutations in *gid* shared by the isolates in each transmission cluster and exclusive to STR resistant clinical isolates (p.Ile-11→Thr, p.Cys-191→Trp, and p.Val-112→Gly). The substitution p.Ile-11→Asn in *gid* was previously associated to STR resistance (Walker et al., 2015; Coll et al., 2018). As Asn and Thr are uncharged polar side chain amino acids the mutation *gid* p.Ile-11→Thr is likely a novel STR resistance determinant. Furthermore, we found genetic variants in *Rv3728* and Cytochrome p450, both previously associated with antimicrobial resistance phenotypes (Raman and Chandra, 2008; Gupta et al., 2010; Kanji et al., 2017, 2019). It will be interesting to in the future further address all these novel candidates at the functional level.

Within the largest transmission cluster of STR resistant isolates (TC1), we found differences in growth in the presence of STR suggesting a continuing process of adaptation. Interestingly, isolates 6C1, 5D1 and 1C8 were genetically indistinguishable in the sense that they were not separated by any high confidence genetic variant. Yet, whether isolates 6C1 and 5D1 showed delayed growth in the presence of a high (10.0 mg/L) STR concentration, isolate 1C8 did not grow at this concentration of the drug. These findings suggest that the phenotype observed in 6C1 and 5D1, and not in 1C8, might be drug tolerance associated with recent or transient genetic variants within the isolates (Wallis et al., 1999; Torrey et al., 2016; Vargas and Farhat, 2020). Drug tolerance mechanisms are elusive, challenging to study and prone to technical- and culture-related artifacts (Lee et al., 2019). Our analysis of low frequency variants in NGS data and Sanger re-sequencing support that 1C8 differs from 6C1 and 5D1 by having one additional cytosine in the homopolymeric cytosine tract of the *glpK* gene. This polymorphism was found in four isolates from the studied cohort including two with low STR resistance. Nonetheless, it was also found in two STR susceptible isolates. Of note, the possible role of glpK in *M. tuberculosis* drug resistance has been a topic of recent debate (Bellerose et al., 2019; Safi et al., 2019, 2020; Vargas and Farhat, 2020). It is thus tempting to speculate that changes homopolymeric cytosine tract of *glpK* may be related to the observed phenotype of low-to-intermediate STR resistance, a hypothesis that will need to be further addressed in future studies (Bellerose et al., 2019; Safi et al., 2019, 2020; Vargas and Farhat, 2020).

The highest level of STR resistance was found in the isolate 2H3 that diverged from the other TC1 isolates by harboring one mutation in the gene *bioF2* (p.Tyr-323→Cys). Although a SNP in an adjacent position of *bioF2* (p.Ile-322→Val) was found in another STR resistant isolate (4E9) from the studied cohort, both genetic variants seem to be rare in unrelated cohorts. The gene *bioF2* is likely involved in *M. tuberculosis* biotin biosynthesis (Salaemae et al., 2011). Mycobacteria rely primarily on *de novo* synthesis of biotin, an important micronutrient serving as an essential cofactor for several enzymes (Park et al., 2011; Salaemae et al., 2011). Consequently, affecting biotin biosynthesis might influence bacilli growth. Thus, *bioF2* p.Tyr-323→Cys, and possibly also p.Ile-322→Val, might influence *bioF2* function and modulate growth in the presence of high concentrations of STR. It will be relevant to test this hypothesis in future experiments using genetically engineered mutants targeting these bioF2 residues. Importantly, the phenotype of high STR resistance of the isolate 2H3 found in *in vitro* cultures was maintained when using the infection model of mouse BMDM in which the bacilli grow within the controlled intracellular microenvironment of the macrophage phagosome.

STR resistance has not been perceived as a major clinical threat since this antibiotic was replaced in first line TB treatment by more efficient combinations of other antimicrobials. However, the hypothesis that STR might predispose to MDR was previously suggested (Buu et al., 2012; Manson et al., 2017; Holt et al., 2018; Bouziane et al., 2019). The mechanisms underlying this process remain elusive, but likely involve secondary mutations that compensate the fitness cost of resistance to more than one drug. *M. tuberculosis* isolates like the ones herein studied probably have the same likelihood to be transmitted as susceptible strains but could represent an additional threat regarding the development of high level STR resistance or even resistance to other drugs. If this hypothesis proves correct, it is of great relevance to survey and increase the efforts to control the transmission of STR resistant strains. In this context, our findings raise concerns on the potential inaccuracy of molecular diagnostic methods to predict STR resistance and the necessity to combine phenotypic and genotypic analysis to generate more comprehensive DR mutation databases. Also, our results propose the modulation of biotin biosynthesis as a novel putative compensatory mechanism for STR resistance.

## Data Availability Statement

The datasets presented in this study can be found in online repositories. The names of the repository/repositories and accession number(s) can be found below: NCBI sequence read archive, SRR13591335-13591460 and European Nucleotide Archive, ERR1193734-1465744.

## Author Contributions

DR, CM, and BC performed experiments. AR, TC, JG, HB, and IC provided essential resources for the study and discussed the manuscript. DR, CM, MS, and NSO analyzed the data. DR prepared the figures, tables, and drafted the manuscript. MS and NSO supervised the study, planned the experiments, and wrote the final version of the manuscript. All authors read and approved the final manuscript.

## Conflict of Interest

The authors declare that the research was conducted in the absence of any commercial or financial relationships that could be construed as a potential conflict of interest.
