# Supplementary material for: Heterogeneous Streptomycin Resistance Level Among Mycobacterium tuberculosis Strains From the Same Transmission Cluster
Source: Front Microbiol. 2021 Jun 11;12:659545. doi: 10.3389/fmicb.2021.659545 (PMC8226182; doi:10.3389/fmicb.2021.659545)
Supplement: Supplementary file 4 [file Image_1.pdf]

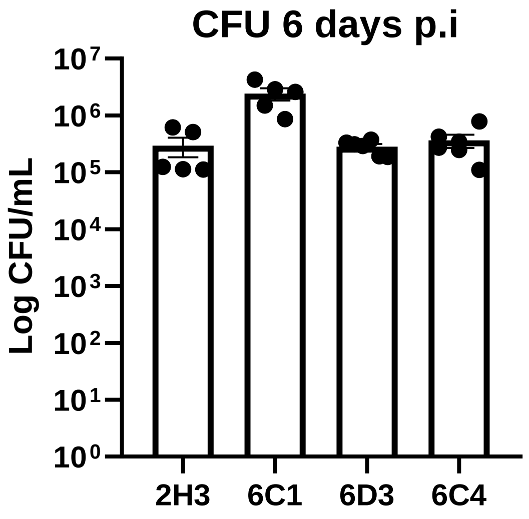

Supplementary Figure 1. Six days post-infection the BMDM cells were lysed and the intracellular bacteria quantified by CFU enumeration.
