# Supplementary material for: Heterogeneous Streptomycin Resistance Level Among Mycobacterium tuberculosis Strains From the Same Transmission Cluster
Source: Front Microbiol. 2021 Jun 11;12:659545. doi: 10.3389/fmicb.2021.659545 (PMC8226182; doi:10.3389/fmicb.2021.659545)
Supplement: Supplementary file 5 [file Image_2.pdf]

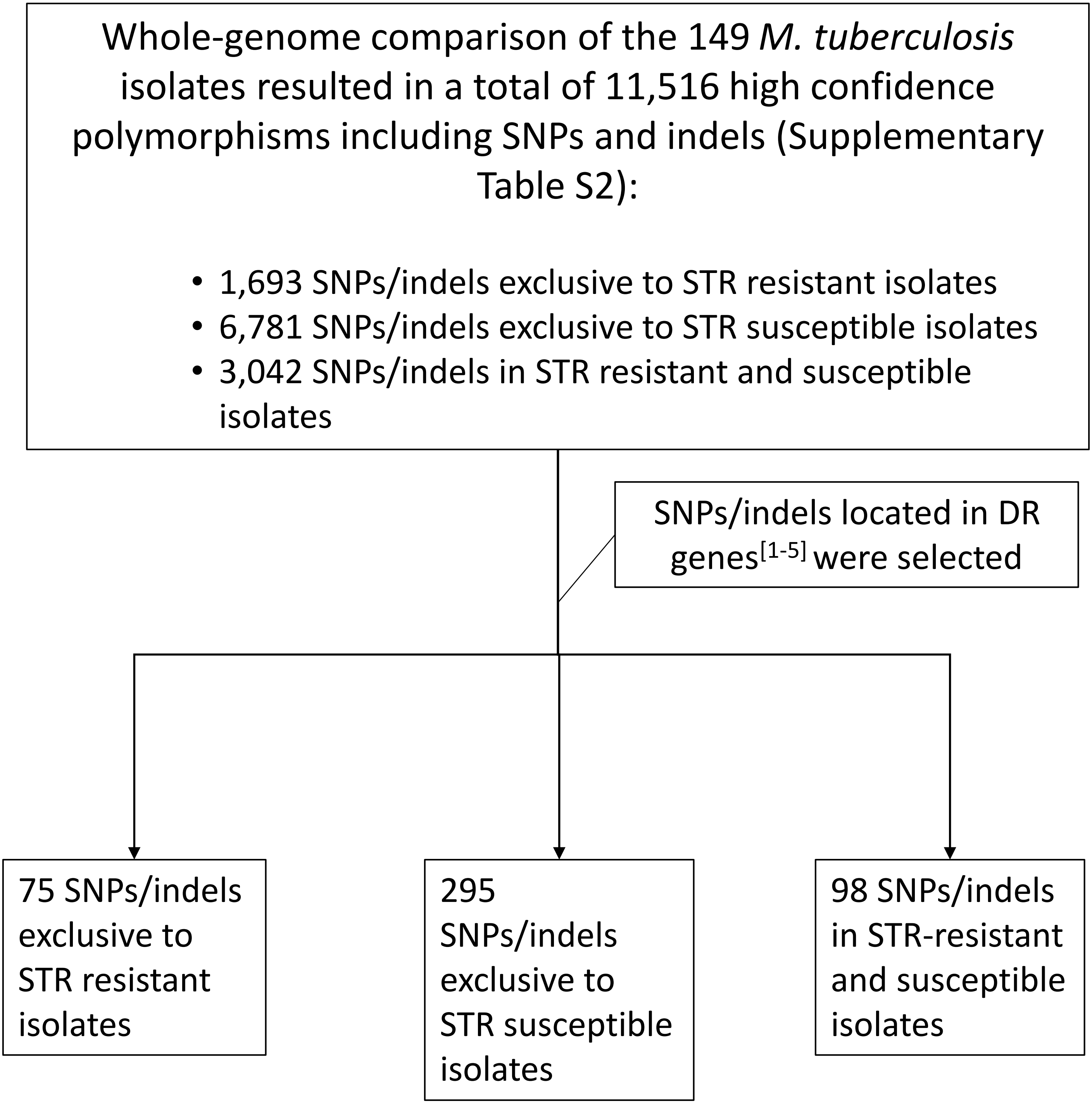

Figure S2: Criteria used to select polymorphisms (SNPs or indels) in genes previously associated with DR and present only in STR resistant *M. tuberculosis i*solates from the studied cohort.

References

1. Raman, K., and Chandra, N. (**2008**). *Mycobacterium tuberculosis* interactome analysis unravels potential pathways to drug resistance. BMC Microbiol. 8, 234. doi:10.1186/1471-2180-8-234.

2. Raman, K., Yeturu, K., and Chandra, N. (**2008**). targetTB: A target identification pipeline for *Mycobacterium tuberculosis* through an interactome, reactome and genome-scale structural analysis. BMC Syst. Biol. 2, 109. doi:10.1186/1752-0509-2-109.

3. Padiadpu, J., Vashisht,R., and Chandra, N. (**2010**). Protein-protein interaction networks suggest different targets have different propensities for triggering drug resistance. Syst. Synth. Biol. 4,311-322. doi:10.1007/s11693-011-9076-5.

4. Kanji, A., Hasan, R., Ali, A., et al. (**2017**). Single nucleotide polymorphisms in efflux pumps genes in extensively drug resistant *Mycobacterium tuberculosis* isolates from Pakistan. Tuberculosis. 107, 20-30. doi:10.1016/j.tube.2017.07.012.

5. Kanji, A., Hasan, R., Hasan, Z., (**2019**). Efflux pump as alternate mechanism for drug resistance in *Mycobacterium tuberculosis*. Indian Journal of Tuberculosis. 1, 20-25. doi: 10.1016/j.ijtb.2018.07.008
