# Supplementary figures and images for: Heterogeneous Streptomycin Resistance Level Among Mycobacterium tuberculosis Strains From the Same Transmission Cluster
Source: Front Microbiol. 2021 Jun 11;12:659545. doi: 10.3389/fmicb.2021.659545 (PMC8226182; doi:10.3389/fmicb.2021.659545)

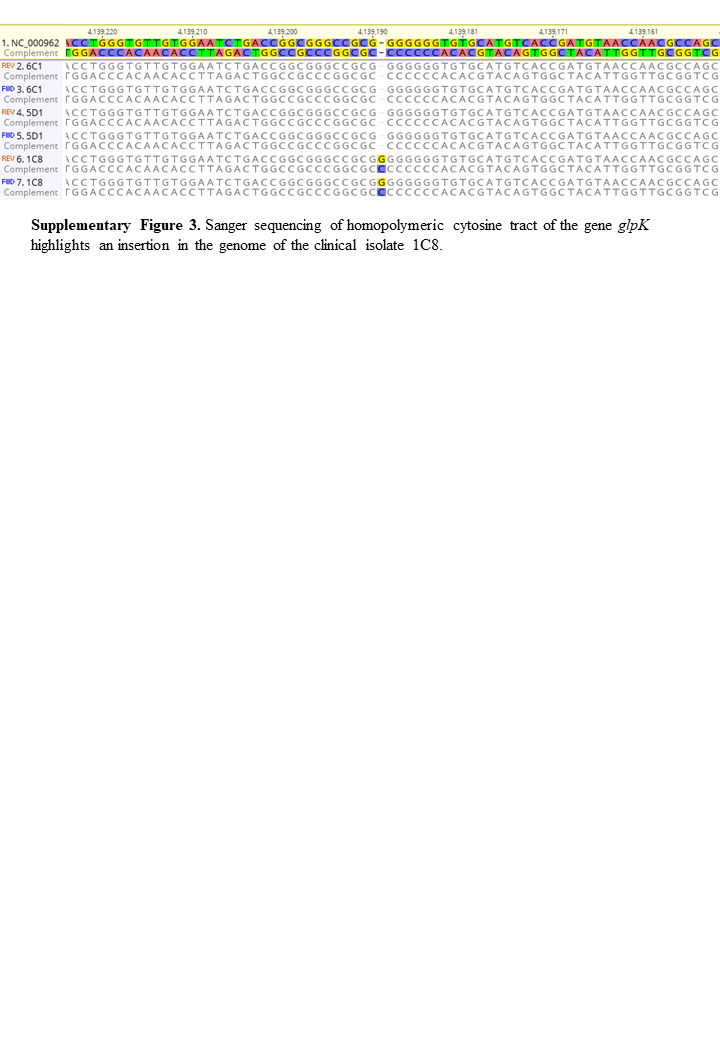

Supplement: Supplementary file 6 [file Image_3.tif]
